# Hypothermic and cryogenic preservation of cardiac tissue-engineered constructs†

**DOI:** 10.1039/d3bm01908j

**Published:** 2024-06-24

**Authors:** Jasmijn Janssen, Nino Chirico, Madison J. Ainsworth, Gerardo Cedillo-Servin, Martina Viola, Inge Dokter, Tina Vermonden, Pieter A. Doevendans, Margarida Serra, Ilja K. Voets, Jos Malda, Miguel Castilho, Linda W. van Laake, Joost P. G. Sluijter, Vasco Sampaio-Pinto, Alain van Mil

**Affiliations:** a Department of Cardiology, Experimental Cardiology Laboratory, Circulatory Health Research Center, Regenerative Medicine Center Utrecht, University Utrecht, University Medical Center Utrecht Uppsalalaan 8 Utrecht 3584 CT The Netherlands A.vanMil@umcutrecht.nl V.M.Sampaiopinto@umcutrecht.nl; b Department of Orthopedics, University Medical Center Utrecht Heidelberglaan 100 Utrecht 3584 CX The Netherlands; c Department of Pharmaceutical Sciences, Utrecht Institute for Pharmaceutical Sciences (UIPS), Utrecht University Universiteitsweg 99 3508 TB Utrecht The Netherlands; d Netherlands Heart Institute (NLHI) Utrecht 3511 EP The Netherlands; e Centraal Militair Hospitaal (CMH) Utrecht 3584 EZ The Netherlands; f iBET, Instituto de Biologia Experimental e Tecnológica Oeiras Portugal; g Instituto de Tecnologia Química e Biológica António Xavier, Universidade Nova de Lisboa Oeiras Portugal; h Department of Equine Sciences, Faculty of Veterinary Sciences, Utrecht University Yalelaan 1 Utrecht 3584 CL The Netherlands; i Laboratory of Self-Organizing Soft Matter, Department of Chemical Engineering and Chemistry & Institute of Complex Molecular Systems, Eindhoven University of Technology Eindhoven 5600 MB PO box 513 The Netherlands; j Department of Biomedical Engineering, Eindhoven University of Technology Eindhoven 5612 AE The Netherlands

## Abstract

Cardiac tissue engineering (cTE) has already advanced towards the first clinical trials, investigating safety and feasibility of cTE construct transplantation in failing hearts. However, the lack of well-established preservation methods poses a hindrance to further scalability, commercialization, and transportation, thereby reducing their clinical implementation. In this study, hypothermic preservation (4 °C) and two methods for cryopreservation (*i.e.*, a slow and fast cooling approach to −196 °C and −150 °C, respectively) were investigated as potential solutions to extend the cTE construct implantation window. The cTE model used consisted of human induced pluripotent stem cell-derived cardiomyocytes and human cardiac fibroblasts embedded in a natural-derived hydrogel and supported by a polymeric melt electrowritten hexagonal scaffold. Constructs, composed of cardiomyocytes of different maturity, were preserved for three days, using several commercially available preservation protocols and solutions. Cardiomyocyte viability, function (beat rate and calcium handling), and metabolic activity were investigated after rewarming. Our observations show that cardiomyocytes’ age did not influence post-rewarming viability, however, it influenced construct function. Hypothermic preservation with HypoThermosol® ensured cardiomyocyte viability and function. Furthermore, fast freezing outperformed slow freezing, but both viability and function were severely reduced after rewarming. In conclusion, whereas long-term preservation remains a challenge, hypothermic preservation with HypoThermosol® represents a promising solution for cTE construct short-term preservation and potential transportation, aiding in off-the-shelf availability, ultimately increasing their clinical applicability.

## Abstract 2

This study is the first to preserve cardiac tissue engineered constructs with encapsulated iPSC-derived cardiomyocytes under hypothermic- and cryogenic conditions.

## Graphical abstract

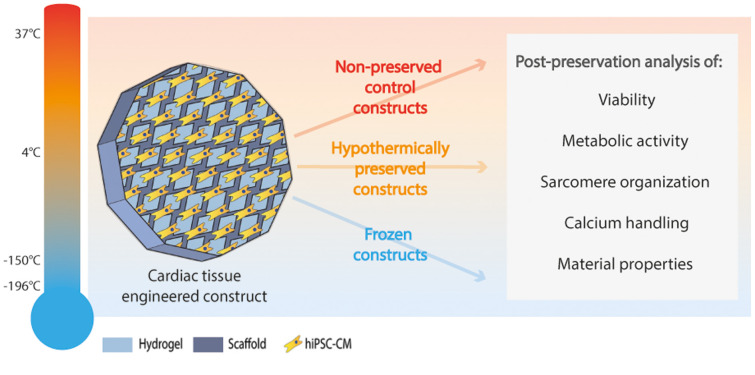

## Introduction

Currently there is no curative treatment for heart failure (HF). The rising number of HF patients (11.8% prevalence in patients above the age of 60 worldwide), associated with improved survival rates after cardiovascular events such as a myocardial infarction (MI), and the scarcity of available transplantable hearts stresses the need for cardiac regenerative therapies such as tissue engineered constructs.^1–6^ Following a MI, the heart is unable to regenerate functional tissue, leading to an irreversible loss of cardiomyocytes and the formation of noncontractile scar tissue. Subsequent adverse remodeling leads to organ dysfunction and eventually to advanced HF.^7,8^ The field of cardiac tissue engineering (cTE) aims to repair and restore the function of the heart post-MI by administering cardiac cells and biomaterials.^9^ Transplantation of cells embedded in biomaterials has shown to enhance cell engraftment and prevent flush-out in comparison to injections of single cells.^10–13^

In recent years, several pre-clinical trials reported the successful implantation of cardiac sheets, leading to long-term cell retention, progressive *in vivo* maturation, and overall improvement of cardiac function.^13,14–17^ As a result, the first clinical trials are being conducted, including phase I (ClinicalTrials.gov identifier: NCT04696328) and phase I/II clinical trials (ClinicalTrials.gov identifier: NCT04396899) in 2020.^18,19^ These trials investigate the feasibility and safety of transplanting cTE constructs onto infarcted cardiac areas of patients with advanced HF.

Despite the promise of cTE, the field currently faces challenges, including a lack of maturation of the implanted cells – that promote potential arrhythmias – a needed human leukocyte antigen (HLA) match to prevent rejection of the graft, and the time required for cTE production.^20–22^ A very practical and logistic problem to date, is a lack of preservation methods that will allow transport between production sites and hospitals to facilitate cTE construct application in different environments and further scalability and commercialization of cTE constructs. These challenges are somewhat comparable to those faced by heart transplantation, which has been partially overcome by *ex vivo* organ preservation methods (up to a maximum of eight hours), ultimately increasing the number of available donor hearts by 15–30%.^23–25^ Reviewing several approaches for short- and long-term preservation has identified two main techniques that hold potential to increase cTE accessibility: hypothermic and cryogenic storage.^20^

Hypothermic storage (subzero to 10 °C) reduces oxygen demand, metabolic activity, and protein synthesis, ultimately slowing down the energy consumption rate, thereby providing a solution for short-term storage of cells, organs, and tissues.^26–28^ To reduce harmful cell processes associated with hypothermic storage, such as ionic imbalance, formation of free radicals, and cytoskeleton damage associated with reduced ATP production, several specialized solutions have been developed, such as University of Wisconsin (UW) solution, histidine-tryptophan-ketoglutarate, Celsior, HypoThermosol® (HTS), and Rokepie (Rp). These solutions work by preventing the depletion of ATP reserves, maintaining ionic, osmotic, and oncotic (protein-induced osmotic pressure) balance, as well as reducing free radicals and providing energy substitutes.^26,28,29^

In turn, cryopreservation stops metabolism entirely by exposing samples to ultra-low temperatures (<−150 °C), hence potentially allowing indefinite storage.^30^ However, during the cooling and rewarming phases, intracellular ice crystals might form, thus damaging tissue and cellular integrity and viability. During cryopreservation, extreme dehydration and intracellular ice-associated damage is prevented by replacing intracellular and extracellular water with cryoprotective agents (CPAs, *e.g.*, glycerol, dimethyl sulfoxide (DMSO)).^31,32^ In cardiac research, cryopreservation efforts have predominantly centred around preserving human induced pluripotent stem cell-derived cardiomyocytes (hiPSC-CMs), including 3D structures, highlighting the practicality and efficacy of this approach.^33^

In this study, we evaluated the effect of three static storage preservation strategies, hypothermic preservation, and cryopreservation following a slow- and fast freezing approach, on a previously established cTE construct. This model consists of 90% hiPSC-CMs and 10% human fetal cardiac fibroblast (hfCFs) embedded in a gelatin and collagen-based hydrogel supported by a polycaprolactone (PCL)-based melt-electrowritten fiber (MEW) scaffold.^34,35^ Post-preservation viability, function, metabolic activity, and morphology were analysed in cTE constructs. Lastly, the effects of preservation on the hydrogel and MEW scaffold physical properties were evaluated (hydrogel swelling behavior, elasticity, surface topography, and crystallinity).

## Experimental section

### Cell culture and differentiation

All experiments were conducted according to the criteria of the code of proper use of human tissue used in the Netherlands and approved by the ethics committees of the University Medical Center Utrecht and Leiden University Medical Center, the Netherlands. Different hiPSCs lines, all deposited in the European Bank for iPSCs (EBiSC, https://ebisc.org/) and registered in the online registry for human PSC lines hPSCreg (https://hpscreg.eu/), were used: UKKi032-C (NP0141-31B) (female) (Fig. S1†), UKKi037-C (NP0144-41) (female), and NP0143-18 UKKi036-C (male).^36^ hiPSCs were differentiated into hiPSC-CMs using a GiWi differentiation protocol.^37^ In short, at day 0, with hiPSCs at 90% confluency, medium was changed to heparin medium (see Table S1† for media compositions) with 4 μM CHIR99021 (Selleck Chemicals). After 48 hours, medium was refreshed with heparin medium with 2 μM Wnt-C59 (Tocris Bioscience). At day 4 and 6, medium was changed with heparin medium. From day 7 onwards, medium was replaced with insulin medium (Table S1†) until purification around day 10. Around day 10, the hiPSC-CMs were beating, and medium was changed with purification medium (Table S1†) every other day until day 15. From day 15 onwards, hiPSC-CMs were expanded following Wnt activation by GSK-3β inhibition with 2.0 to 4.0 μM CHIR99021 and the inhibition of cell–cell contact.^38,39^ Human fetal cardiac fibroblasts (hfCFs) (P6-10) were isolated and cultured as described previously.^40^

### Construct preparation

#### Melt electrowriting (MEW) scaffold fabrication

MEW microfiber scaffolds were fabricated as previously described.^34,35,41^ Briefly, hexagonal MEW scaffolds with a side length of 400 μm were fabricated using a 3DDiscovery (regenHU) and medical-grade PCL granules (Purasorb, Corbion). Constructs with an 8 mm diameter and ∼400 μm in thickness comprised 20 layers with a fiber diameter of 11 μm. For tensile analysis 5 × 10 mm constructs were prepared.

#### Construct seeding

The cell suspension was resuspended in a blend of 5% w/v porcine skin gelatin methacryloyl (GelMA, Sigma-Aldrich, G2500) (80% degree of functionalization) and 0.8 mg mL^−1^ type 1 rat tail collagen (RatCol®) supplemented with 0.5 mM tris (2,2′-bipyridyl)dichlororuthenium(ii) hexahydrate (Ru, Sigma-Aldrich, 544981) and 5 mM sodium persulfate (SPS, Sigma-Aldrich, S6172) as photo-initiators^42^ and casted within a hexagonal PCL scaffold (thickness ∼400 μm, diameter = 8 mm).^34^ Crosslinking was initiated with white light (floodlight) for 4 minutes. Subsequently, the constructs were incubated at 37 °C for collagen thermal crosslinking for 12 minutes.^35^ 75% of the construct culture media (Table S1†) was changed every other day.

### Preservation methods

Hypothermic preservation was conducted on a slowly moving (approx. 15 rpm) rocking platform (Polymax 1040, Heidolph Instruments) at 4 °C for 3 days. Rokepie-FD01® (Rp), HypoThermosol® (HTS), and CoStorSol®/University of Wisconsin (UW) were utilized according to manufacturer instructions (Table S2†). Plates were sealed air-tight with parafilm, preventing a drop in CO_2_ to cause an increase in the solution's pH.^43,44^ Standard cryopreservation following a slow freezing protocol was initiated by transferring the construct into a cryovial (Cryo.S™) with 1 mL pre-cooled standard freezing media (4 °C) (Table S2†) and cooled and stored for one day in a CoolCell container (Biocision) at −80 °C with a cooling rate of −1 °C min^−1^. After 24 hours, the vial was transferred to a liquid nitrogen freezer (−196 °C), where it stayed for an additional 2 days (3 days of cryogenic preservation in total). Fast freezing of cTE constructs was conducted in aluminium cryotubes (Sanbio). Constructs were incubated in 1 ml pre-cooled (4 °C) standard freezing media (Table S2†) for 30 minutes to allow DMSO permeation.^45,46^ Following incubation, the cryotube was carefully dipped in a −80 °C isopropanol bath for 2.5 minutes. During these 2.5 minutes, the constructs reached an average temperature of −60 °C, with freezing rates ranging from >150 °C min^−1^ during the first seconds to 11.4 °C min^−1^ from −50 °C to −60 °C as measured with a temperature logging digital thermometer (Fluke, 53 IIB) (Fig. S2†). Thereafter, cryotubes were transferred to a −150 °C freezer, where they were preserved for 3 days. Post-preservation, conventional thawing in a 37 °C water bath was performed as recommended for samples <2–3 mL.^31^ Briefly, samples were submerged in the water bath until only a sliver of ice remained (15 s) and constructs were transferred to a 24-well plate filled with 2 mL pre-warmed construct media. Media was refreshed one hour and one-day post-thawing, followed by media changes every other day.

### Method of time matching of controls and preserved constructs

**Fig. 1:**
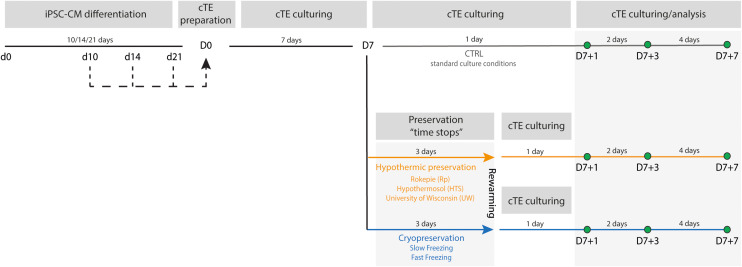
Experimental timeline. Visual representation of the incorporation of hiPSC-CMs with different ages (d10, d14 and d21, black dotted lines) into the cTE constructs and time-matching of the controls and preserved constructs. After seven days of cTE construct culturing, the constructs were divided into three groups, subjected to the different experimental conditions. Control constructs (grey line) were kept under standard cTE culture conditions during the experiments. Preserved constructs were subjected to 3 days of hypothermic- (orange line) or cryogenic (blue line) conditions. Constructs were analyzed at three different timepoints: D7 + 1, D7 + 3 and D7 + 7.

For each experiment, cTE constructs were prepared and cultured for 7 days. Following this first week, constructs were randomly allocated to one of the experimental groups: hypothermic preservation for 3 days, cryogenic preservation for 3 days or control culture conditions for 1 day. Due to the low temperatures achieved during preservation, construct metabolism and maturation are stopped for 3 days. After 3 days of preservation, the constructs were rewarmed by transferring them to pre-warmed 37 °C fresh construct medium. After a recovery period of 24 hours, preserved constructs were analysed and compared to their time-matched controls (CTRL) (Fig. 1). Analysis timepoints are denoted as D7 + *X*, representing the age of the constructs (7 days of culturing + *X* days of culture under standard culture conditions after allocation to their experimental conditions). For instance, D7 + 1 control constructs were cultured continuously for 8 days, while D7 + 1 preserved constructs were cultured for 7 days, preserved for 3 days (during which time stopped), and cultured for one more day to allow for cTE recovery. This time-matching strategy ensured that both controls and experimental groups (hypothermic or cryogenic preservation) were compared after spending an equal time in culture at 37 °C, when cTE metabolism and functionality is highest.

### Evaluation of cell viability – LIVE/DEAD analysis

Cellular viability was determined with the LIVE/DEAD viability/cytotoxicity kit (ThermoFisher Scientific, L3224) following manufacturer instructions. The stained constructs were visualized and captured in three different locations – once in the center and twice at the construct periphery – *via z*-stack imaging with Leica Thunder microscopy (*z*-stack size = 102.94 μm) (Fig. S3†). Maximum projections were obtained from the equal-sized *z*-stacks and cell viability was quantified by determining the area of red and green fluorescence (dead/live cells, respectively) on the maximum projection images using the ImageJ software. Data were normalized to time-matched CTRL.

### Beating rate

Twenty-second videos were taken using a GoPro Black Hero 7 camera connected to a bright field microscope (Olympus CKX41) *via* a c-mount system (Olympus U-TV1X-2). During video acquisition, cTE constructs were kept at 37 °C using a warm base plate.

### Cellular metabolic activity, metabolic substrate quantification, and high-sensitivity troponin assay

Cellular metabolic activity was evaluated with the alamarBlue cell viability assay (Thermo Fisher Scientific, DAL1025) following manufacturer instructions. In short, 10% alamarBlue solution in construct medium was added to the constructs. After 4 hours, the reacted solution was transferred to a 96-well plate and fluorescence was recorded using a fluorescence excitation wavelength of 560 nm and an emission wavelength of 590 nm. Absorbance was monitored at 570 nm and presented in arbitrary fluorescent units.

Conditioned culture media was collected at different time points (*i.e.* before preservation, at D7 + 1, D7 + 3, and D7 + 7 post-preservation) and analyzed using an Atellica CH Analyzer (Siemens). Glucose (Atellica CH Glucose Hexokinase_3; REF 11097592), lactate (Atellica CH Lactate_2; REF 11532568), and cardiac troponin-I levels (Atellica IM High-Sensitivity Troponin I; REF 10997840) were measured. To prevent media change-associated differences between different timepoints, media was always refreshed one day prior to media collection for analysis.

### Calcium handling

Three days post-preservation (D7 + 3), when preserved cTE constructs re-start contracting (as individual hiPSC-CMs or as small or larger clusters), calcium handling was evaluated. cTE constructs were incubated with construct culture media with 25% fluorbright DMEM (ThermoFisher Scientific, A1896701) supplemented with 1 : 1000 Cal-520® AM (Abcam, ab171868) and 8% Pluronic F-127 (Sigma-Aldrich) for 45 minutes at 37 °C. Thirty second videos were taken at three different locations – once near the centre and twice at the periphery of the construct – with a Leica Thunder microscope (Fig. S3†) and as previously described.^33^ Data analysis was performed using FIJI and Peaks, a custom-written Matlab script (https://doi.org/10.17605/OSF.IO/86UFE).

### Evaluation of post-preservation hydrogel properties

The effects of preservation on the hydrogel were determined three days post-preservation (D7 + 3) *via* a sol-fraction analysis, percentage of macromer, and the mass swelling ratio. *Hydrogel sol-fraction analysis*: sol-fraction was performed using the protocol from Blocki *et al.*^47^ Percentage of macromer, mass swelling ratio (*q*), and the sol-fraction could be determined following the equations below:1
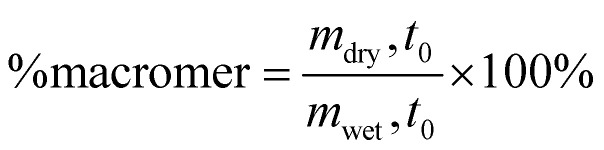
2
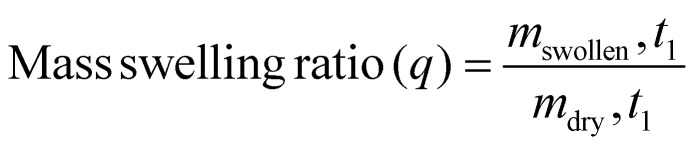
3
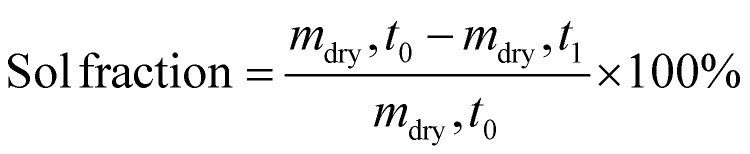


### Evaluation of post-preservation PCL scaffold properties

Preserved PCL scaffolds were analyzed three days post-preservation (D7 + 3) by scanning electron microscopy (SEM), differential scanning calorimetry (DSC), and tensile stress tests and compared to non-preserved controls. SEM pictures were taken of all groups (*n* = 3), using a backscatter detector with 10 kV acceleration voltage after pre-coating the constructs with a 2 nm gold coating to enhance conductivity. To assess thermophysical properties, CTRL, HTS and fast frozen constructs were analyzed using DSC Q2000 equipment (TA instruments). A cycle of scans (heating–cooling–heating) was performed on the PCL scaffolds (5 mg, loaded into Tzero aluminium pans (TA Instruments)) from 0 to 150 °C with a heating rate of 10 °C min^−1^ and a cooling rate of 1 °C min^−1^, under nitrogen flow of 50 mL min^−1^. Melting temperature (*T*_m_) (°C), enthalpy (J g^−1^), and crystallinity were determined for both groups of preserved constructs and compared to controls. Melting temperatures (*T*_m_) were determined from the onset of the endothermic peaks. Lastly, uniaxial tensile tests were performed with a 25 N force gauge in a Multitest 2.5-dV mechanical tester (Mecmesin), and data was recorded using VectorPro software (Mecmesin).

### Statistical analysis

All statistical analyses were performed in GraphPad Prism v9.4.1 (GraphPad Software Inc., La Jolla, USA) and data expressed as mean ± standard deviation. Ordinary one way or two-way analysis of variance (ANOVA) with Tukey's multiple comparisons were carried out for all analyses. For the analysis of the calcium transients, a multiple comparison Kruskal–Wallis test was performed. For all analyses, a *p*-value less than 0.05 was considered to be significant.

## Results

In this study, long- and short-term preservation techniques and solutions were tested on cTE constructs.^34,35^ The cTE construct represents a complex system with multiple cell types and biomaterials, where cell viability and function are linked to hydrogel and scaffold integrity.^34,35^ This relationship needs to be maintained regardless of the preservation strategy; thus, a wide set of parameters are investigated, at both the cellular and material level.

### HypoThermosol® and fast freezing best preserved cTE constructs viability

To determine the impact on and feasibility of preservation of cTE constructs, three commercially available hypothermic preservation solutions were tested: ROKEPIE (Rp), University of Wisconsin solution (UW), and HypoThermosol® (HTS). Additionally, two cryopreservation methods were applied: slow- and fast freezing (Fig. 1).

**Fig. 2:**
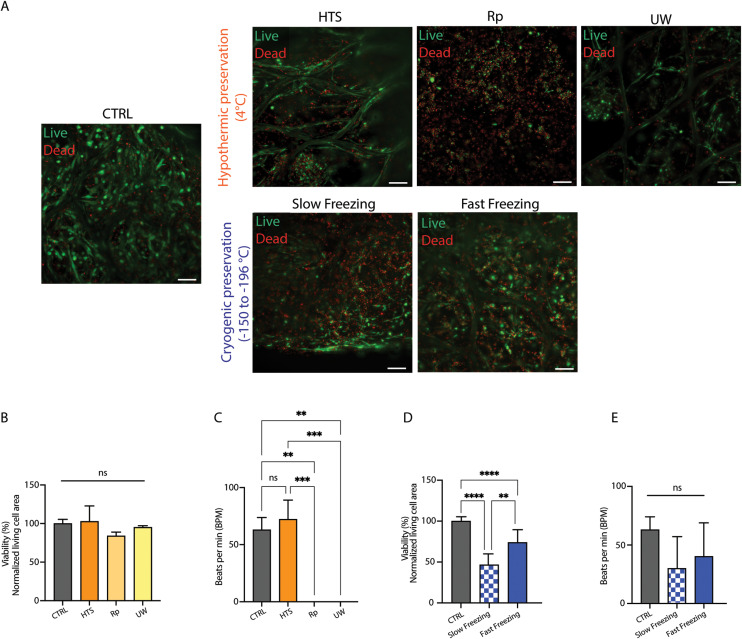
HypoThermosol® and fast freezing best preserved short- and long-term cTE construct viability. (A) Live/dead imaging 3 days after rewarming (D7 + 3). Living cells, (green/calcein-AM positive), and dead cells (red/ethidium homodimer-1 positive). Channels are overlaid. Scale bar = 150 μm. Representative images are shown. (B) Live/dead quantification, normalized to time-matched unpreserved CTRL. CTRL = 100% ± 5.4%, HTS = 102.9% ± 20%, Rp = 84.0% ± 4.9%, and UW = 95.2% ± 2.0% (median age: d14, *n* = 14, 13, 2 and 2, respectively). (C) Beating rate of cTE constructs preserved under hypothermic conditions. HTS showed signs of functional decline (Video S2†) while no contraction was detected in the Rp and UW groups. (D) Live/dead quantification, normalized to time-matched unpreserved CTRL. CTRL = 100% ± 5.0%, Slow freezing = 48.7% ± 12.5%, and fast freezing = 73.4% ± 15.7% (median age: d21, *n* = 16, 4, and 16, respectively). (E) Beating rate of cTE constructs subjected to slow- and fast freezing (Videos S3 and S4†). A non-significant decrease was observed in preserved constructs compared to time-matched unpreserved CTRL.

Seven-day-old cTE constructs were preserved for three days in hypothermic solution (at 4 °C) and subsequently rewarmed and cultured for seven days (Fig. 1 – orange line). Viability analyses at D7 + 3 showed that HTS exhibited a trend of higher cell viability (102.9% ± 20%) compared to other solutions (Rp: 84.0% ± 4.9%, and UW: 95.2% ± 2.0%) and the time-matched unpreserved constructs (CTRL: 100% ± 5.4%) (Fig. 2A and B). After rewarming, cTE constructs preserved in the presence of Rp and UW did not resume contractions, while those preserved in HTS showed no signs of functional decline, with a synchronized beating rate comparable to the unpreserved CTRL (Fig. 2C) (Videos S1 and S2†).

Slow- and fast freezing resulted in a significant decrease in construct viability and functional impairment at D7 + 3. Slow freezing resulted in 48.7% ± 12.5% survival while fast freezing ensued 73.4% ± 15.7% viability after rewarming (Fig. 2D and E). Of note, the beating rate showed a non-significant decrease in cryopreserved constructs compared to CTRL (Fig. 2E and Videos S1, S3, S4†). However, preserved constructs displayed patchy, unsynchronized beating clusters. This effect was even more pronounced in the slowly frozen constructs (Video S3†) that had fewer beating clusters compared to the fast frozen group. Furthermore, after a longer, 7-days of culture after rewarming, no beating clusters could be observed in the slowly frozen constructs, while beating clusters could still be distinguished in fast frozen constructs.

Overall, these results indicate that HTS-based hypothermic preservation and fast freezing were most effective at maintaining cTE construct viability and contractility. For this reason, these conditions were selected for subsequent experiments.

### Cardiomyocyte maturation does not influence the viability of cTE constructs

The differentiation day or maturity of cardiomyocytes has been shown to influence their post-preservation viability and functionality in a 2D setting, emphasizing the possibility of an effect of cardiomyocyte age in the context of cTE construct manufacturing and recovery yields after preservation.^48–50^ We investigated the influence of cardiomyocyte maturation on post-preservation viability and beating behaviour following the most promising preservation approaches: HTS and fast freezing.

In this study, we evaluated hiPSC-CM age-dependent recovery by preparing cTE constructs with hiPSC-CMs at differentiation day 10 (d10, initial beating), day 14 (d14, replating day), and day 21 (Fig. 1, black dashed lines). Time-matched controls (CTRL) from the same construct batch were kept in culture for comparison (Fig. 1, grey line). Post-preservation, viability was determined at D7 + 1 and D7 + 3, one and three days after rewarming, respectively (Fig. 1).

**Fig. 3:**
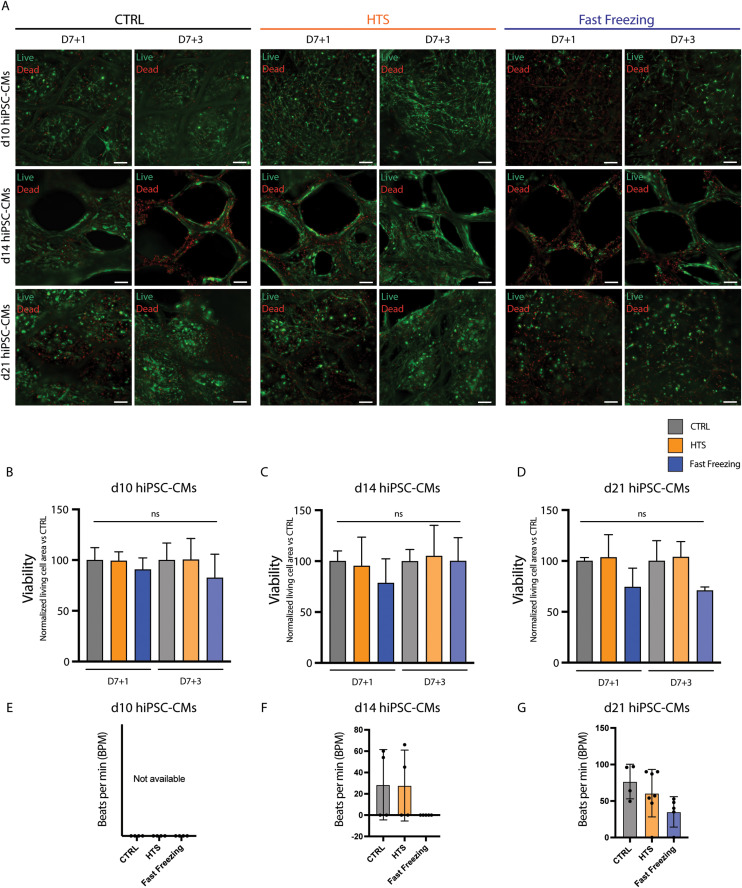
Cardiomyocyte age does not influence viability of cTE constructs. (A) Live/dead imaging at 1 (D7 + 1) and 3 (D7 + 3) days after rewarming. Living cells, (green/calcein-AM positive), and dead cells (red/ethidium homodimer-1 positive). Channels are overlaid. Scale bar = 150 μm. Representative images are shown. (B–D) Live/dead quantification, normalized to time-matched unpreserved CTRL for d10, d14 and d21 hiPSC-CMs (*n* = 4). (E–G) Beating rate of cTE constructs with d10, d14, and d21 hiPSC-CMs at D7 + 3 (Videos S8–S13†). The incorporation of d10 iPSC-CMs did not yield contractile cTE constructs. No significant differences were observed between preserved constructs and CTRL.

Live/dead analysis showed no significant differences between CTRL and preserved samples, irrespective of the analyzed timepoint and hiPSC-CMs age (Fig. 3A and B). However, cardiomyocyte age greatly influenced cell organization and the contractile phenotype of cTE constructs. Constructs prepared with d21 hiPSC-CMs displayed synchronous beating of the whole construct before preservation (D7) (Video S7†). In contrast, pre-preservation constructs prepared with d10 or d14 hiPSC-CMs often showed no or cluster-like contractions and different tissue morphology (Videos S5, S6,† and Fig. 3A). At D7 + 3, d10 hiPSC-CM constructs showed no signs of contraction, d14 hiPSC-CM constructs showed irregular beating behavior or no contraction (Videos S8–10†), and d21 hiPSC-CM constructs showed more consistent beating behavior across all experimental groups (Videos S11–S13†).

Altogether, and in line with previous observations, d21 hiPSC-CMs have a greater ability to re-arrange the supporting hydrogel and connect with each other before preservation, and do not show lower viability compared to younger cells, establishing the basis for subsequent experiments using d21 hiPSC-CMs.^35^

### HypoThermosol® and fast freezing reduced cTE construct metabolism and recovery post-preservation

**Fig. 4:**
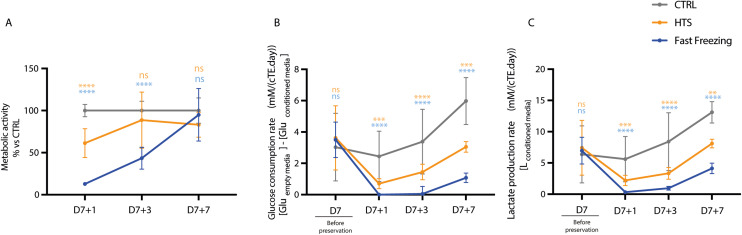
Hypothermosol® and fast freezing reduced cTE construct metabolism and recovery post-preservation. (A) Redox activity of preserved constructs determined by the Alamar Blue assay at days 1 (D7 + 1), 3 (D7 + 3) and 7 (D7 + 7) after rewarming; HTS-preserved (*n* = 7), fast frozen (*n* = 7) CTRL (*n* = 8). (B) Glucose consumption rate of hypothermically preserved (HTS, *n* = 20) and fast frozen (*n* = 18) constructs, was compared to that of unpreserved controls (CTRL, *n* = 16) at days 1 (D7 + 1), 3 (D7 + 3) and 7 (D7 + 7) after rewarming. (C) Lactate production of HTS-preserved (*n* = 20), fast frozen (*n* = 18) and CTRL (*n* = 16) constructs was compared pre-preservation and at days 1 (D7 + 1), 3 (D7 + 3) and 7 (D7 + 7) after rewarming. **p < 0.01, ***p < 0.001, ****p < 0.0001, and ns indicates a non-significant difference. The color indicates whether the comparison was made between CTRL *vs*. HTS (orange) or CTRL *vs*. fast freezing (blue).

The impact of HTS and fast freezing on the metabolic activity of cTE constructs were investigated by measuring cellular redox activity, glucose consumption, and lactate production. cTE constructs subjected to fast freezing displayed an acute reduction of metabolic activity (12.82% ± 1.33%) at D7 + 1, compared to CTRL. Fast frozen constructs’ metabolic activity gradually increased over time to 43.44% ± 13.04% on D7 + 3 and 94.98% ± 28.18% on D7 + 7. After hypothermic preservation, the metabolic activity of HTS-preserved constructs initially dropped to 61.26% ± 17.13% on D7 + 1 but was no longer distinguishable from that of CTRL constructs from D7+3 onwards (Fig. 4A).

The iPSC-CMs and hfCFs embedded in the cTE constructs consume metabolic substrates like glucose from the medium, while lactate is released as a by-product of glucose consumption.^51^ Before preservation (D7), comparable glucose consumption (3.03 mM to 3.63 mM) and lactate production (6.37 mM to 7.43 mM) were observed among experimental groups. After preservation and rewarming, on D7 + 1 and D7 + 3, fast frozen constructs presented no appreciable glucose consumption or lactate production. Limited recovery was subsequently observed at D7 + 7 with glucose consumption increasing to 1.08 mM ± 0.30 mM and lactate production to 4.13 mM ± 0.84 mM. On the contrary, HTS constructs showed signs of recovery at D7 + 3, for both glucose and lactate, but failed to reach the levels measured in CTRL (Fig. 4B and C). These results indicate that the metabolic activity of cTE constructs is strongly inhibited after preservation, particularly in the fast frozen group, though partial recovery occurs during culture after rewarming in the HTS group.

### The impact of HTS and fast freezing on hiPSC-CMs sarcomere organization and calcium handling

**Fig. 5:**
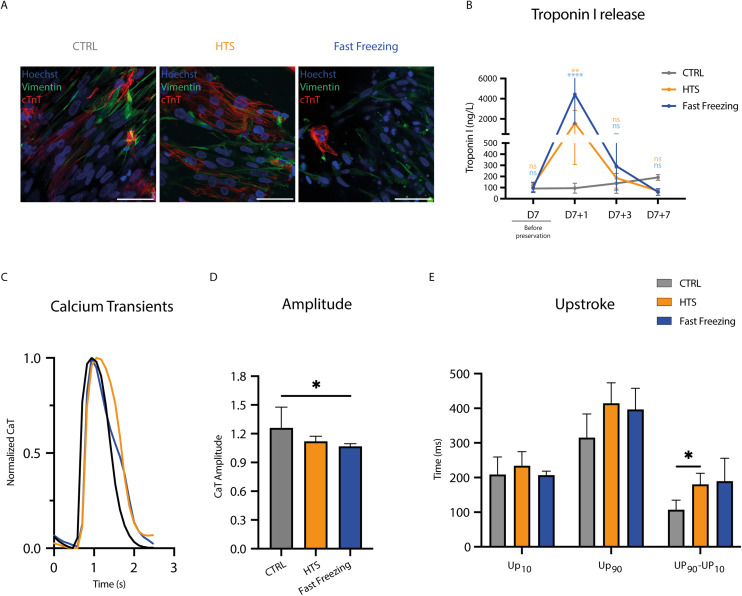
The impact of HTS and fast freezing on hiPSC-CM sarcomere organization and calcium handling. (A) Representative immunofluorescence images taken at D7 + 3. CTRL and HTS showed comparable presence of well-arranged and dense sarcomere protein expression (cardiac troponin T (cTnT)). Fast frozen constructs showed reduced cTnT expressing cells. Scale bars = 200 μm. (B) Troponin-I release to the medium (*n* = 3 to 13). (C) Representative normalized calcium transients of constructs at D7 + 3. (D) Calcium transient amplitude at D7 + 3. (E) Calcium transient upstroke kinetics at D7 + 3.

The damage imposed by cryo- and hypothermic preservation resembles reperfusion post-MI, including a loss of sarcomere content, arrangement, and functionality, resulting in disrupted contractility and calcium handling, both critical for cTE construct performance.^25,48,52^ Following fast freezing, D7 + 3 cTE constructs showed a strong reduction in troponin-T expressing cells compared to CTRL and HTS, which showed a comparable amount (Fig. 5A). Additionally, at D7 + 1 troponin-I release to the medium was substantially higher in fast frozen constructs (4428.1 ng mL^−1^ ± 2925.59 ng mL^−1^), and to a lesser extent in HTS cTE constructs (1576.47 ng mL^−1^ ± 1267.42 ng mL^−1^), compared to CTRL (91.33 ng mL^−1^ ± 26.73 ng mL^−1^). Follow-up measurements (D7 + 3 and D7 + 7) showed no differences between HTS, fast freezing or CTRL (Fig. 5B). Calcium handling at D7 + 3 showed a decreased CaT upstroke velocity for both HTS and fast frozen samples (Fig. 5C–E), with fast freezing displaying a slight decrease in CaT amplitude (Fig. 5D) and no propagation between cell clusters, clearly highlighting the damage in the fast frozen samples (Videos S14–S16†).

### Effect of preservation on the mechanical properties, surface topography and crystallinity of cTE constructs

**Fig. 6:**
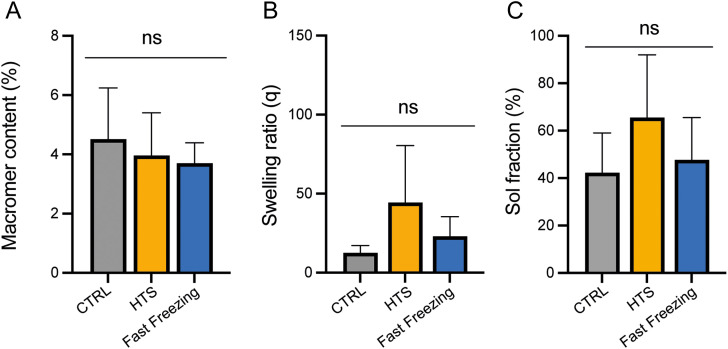
The effect of hypothermic preservation and fast freezing on the cell-supporting hydrogel at D7 + 3. (A) Macromer percentage, (B) mass swelling ratio, and (C) sol fraction. *n* > 4.

Upon implantation, cTE constructs need to withstand the continuous contractions and relaxation of the myocardium. However, as recently reported, rapid cooling/warming of polymers, particularly filaments, can lead to changes in mechanical properties due to alterations in crystallinity.^20,53^ First, the effect of hypothermic preservation and fast freezing on the cell-supporting hydrogel was investigated. The results reveal no significant differences in the macromer percentage, mass swelling ratio, and sol fraction (Fig. 6A–C).

**Fig. 7:**
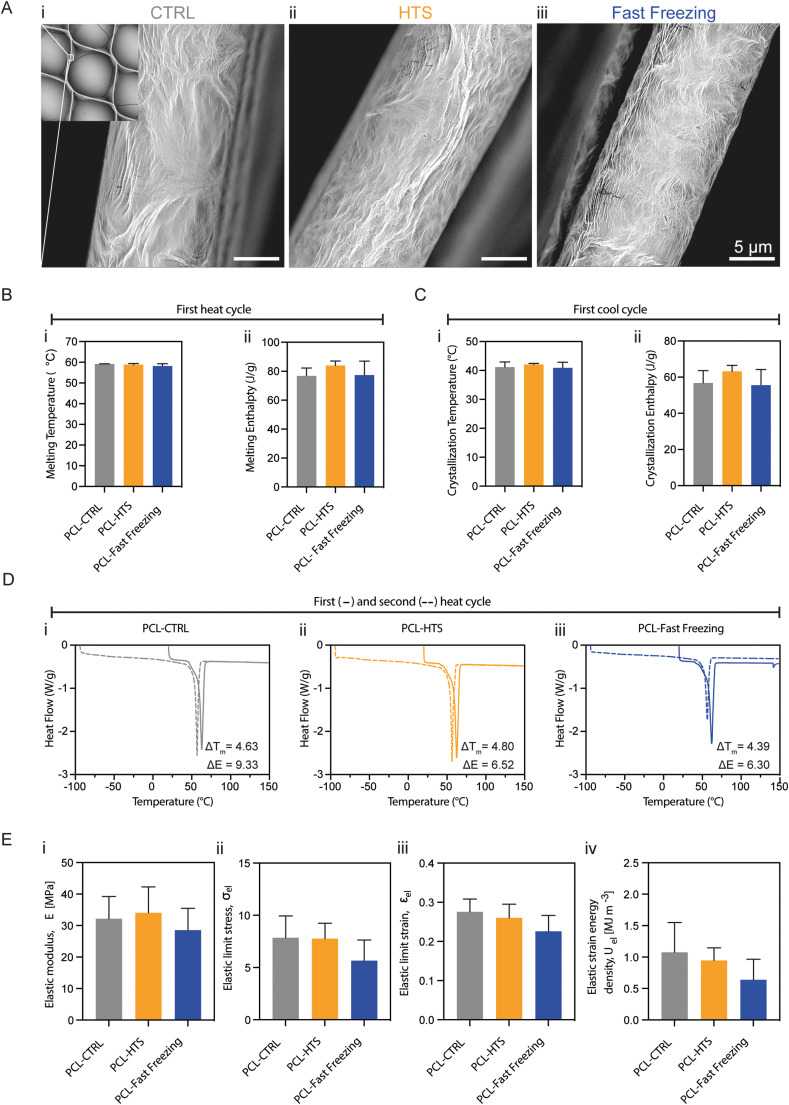
No significant changes to scaffold mechanical properties, surface topography, and crystallinity of cTE constructs. (A) Scanning electron microscopy images of scaffold fibers at D7 + 3. Top-left corner a wide overview of the scaffold. Representative images are shown. *n* = 4. (B–D) Differential scanning calorimetry analysis of samples. (B) Melting temperature and enthalpy from first heating ramp. Data shown as mean with standard deviation as error bars. *n* > 3. (C) Crystallization temperature and enthalpy from first cooling ramp. Data shown as mean with standard deviation as error bars. *n* > 3. (D) First and second heating ramps plotted per condition with calculated mean change of melting temperature and enthalpy. *n* > 3. (E) Tensile mechanical analysis of samples with calculated elastic modulus, elastic limit stress, elastic limit strain and elastic strain energy density. Individual data points plotted with mean and standard deviation as error bars. *n* = 6.

Additionally, fast freezing may shrink the polymer filaments leading to cell detachment and, ultimately, apoptosis.^20,53^ Concurring with this, live/dead imaging of fast frozen cTE constructs revealed the presence of several dead cells particularly in the vicinity of PCL fibers (Fig. S4†). SEM was used to closely observe the topography of the PCL mesh, but no lasting impact on the rewarmed PCL fibers was observed for either of the two preservation methods (Fig. 7A). Further investigation into PCL network remodeling after preservation focused on its melting curves and crystallinity. DSC measurements reveal how the first heating cycle, which may be influenced by the thermal history of the polymer, presented readings of melting temperature and melting enthalpy that were not statistically different between CTRL, HTS and fast frozen constructs (Fig. 7B). Similarly, the crystallization temperature and enthalpy were essentially the same (Fig. 7C). Comparably, similar trends in the change of enthalpy and melting temperature between the first and second heat cycles were observed in all the groups (Fig. 7D). Finally, analysis of bulk mechanical properties of the PCL scaffold through uniaxial tensile testing showed no significant differences in the elastic stress limit and the elastic strain energy density (Fig. 7E).

## Discussion

Despite numerous advances in cTE, several hurdles limit the application of cell-based cTE constructs in the clinical arena. Recent advances in HLA-compatible hiPSC lines would allow the production of cTE constructs upfront, thus making them readily available for biobanking, and implantation as an off-the-shelf treatment for acute patients.^54^ Thus, efficient preservation and off-the-shelf availability has the potential to revolutionize heart failure treatment by extending the time between production and implantation. Yet, effective preservation methods for cTE constructs are currently lacking, ultimately hindering their scalability, commercialization, and transport. Finding solutions to this challenge will increase the accessibility of tissue engineered approaches and their potential benefits to patients suffering from several diseases not only limited to the heart.^20^

This study aimed to identify the optimal method for preserving a complex cTE construct. This construct consisted of hiPSC-CMs and hfCFs, seeded in an ECM-mimicking hydrogel composed of GelMA-collagen,^55^ and embedded in a PCL-based MEW microfiber scaffold with hexagonal micro-architecture to promote cellular organization and improved contraction.^34,35,56^ Hence, the cTE constructs used herein represent a large group of cardiac constructs currently under development as disease models or clinical therapy.

The goal of short-term preservation, at hypothermic temperatures, is to maintain construct viability for a few days (three days in this study) to allow for safe transportation. By transporting tissue under hypothermic conditions, one avoids the build-up of gas and stress factors, the depletion of nutrients and growth factors, and the pH changes associated with the secretion of lactic acid and other waste products after >48 hours of transport at 37 °C.^57^ For this application, HTS yielded superior post-preservation recovery among the three commercially available solutions tested. After rewarming, HTS-preserved constructs showed less functional decline, with a beating rate comparable to that of unpreserved CTRL, whereas Rp and UW did not recover contractility after rewarming (Fig. 2C). These results are analogous with previous studies were hiPSC-CMs preserved in HTS as monolayers or aggregates showed a metabolic activity ranging from 60 to 70% after 7 days of storage.

HTS outperforming Rp and UW could be linked to its broader range of action compared to RP and UW.^58,59^ HTS acts as a PH buffer, ROS scavenger, energy substrate, and ion, osmotic, and oncotic balancer. Whereas UW only ensures osmolarity and reduced oncotic damage by scavenging free radicals, and Rp increases mitochondrial respiration, thus counteracting slower metabolism.

The goal of long-term preservation at cryogenic temperatures is to maintain construct viability for a hypothetical indefinite span of time. This would ensure the biobanking and effective off-the-shelf availability of cTE constructs. In our model, fast freezing outperformed slow freezing. However, both methods critically impaired constructs viability and functionality. These findings are in line with previous experiments with cardiac aggregates, small tissues, and whole hearts.^60,61^ The superiority of fast freezing compared to slow freezing could be explained by the faster cooling rates partly protecting the constructs from irremediable damage caused by cell dehydration during the cooling phase. During slow freezing, osmotic pressure forces water to exit from the cells. If cooling rates are fast enough, water efflux cannot occur before freezing. Consequently, the water becomes trapped within the cells, limiting damage caused by cell dehydration and shrinkage.^31,32,62^

Interestingly, hiPSC-CM maturation status did not have a significant effect on cTE construct viability post-preservation for both HTS or fast freezing (Fig. 3A–D). This contrasts with previously published research on 2D cultures of hESC-CMs, reporting better results after freezing hESC-CMs pre-beating stage, compared to post-beating stage.^28,48,49^ However, we observed that incorporating more mature hiPSC-CMs resulted in synchronous beating, extending throughout the whole scaffold before preservation (D7) and showed improved cell and matrix re-arrangement (Fig. 3A), as reported in previous publications.^35^ Moreover, storing more mature constructs led to improved preservation of beating characteristics (Fig. 3E and F). In light of these findings, the decision was made to proceed with the preservation of constructs made with d21 hiPSC-CMs.

Characterizing the impact of preservation on viability in complex 3D structures is often challenged by the inability of assessing the full depth of the sample, potentially leading to selection bias. Herein, LIVE/DEAD quantifications were conducted in large *z*-stacks, that were maintained constant across different experiments and experimental groups, thus providing a good and unbiased overview of cTE viability throughout the construct. In addition, beating rate measurements, used to characterize cTE constructs functionally, are often not sufficient and can yield misleading results. In fact, similar beating rates were observed in preserved and unpreserved constructs, but the underlying dynamics varied significantly, as shown in ESI Videos 1–13.† While CTRL constructs show often a cohesive, synchronized rhythm throughout the whole construct, HTS and cryopreserved constructs mostly display clustered- or single cell beating, respectively. Therefore, the inclusion of representative videos alongside beating rate data is indispensable for providing a comprehensive understanding of the complex dynamics underlying cTE constructs.

Recent studies have described the damage imposed by cryo- and hypothermic preservation to be similar to that caused by reperfusion after an MI, including oxidative stress, damage-associated and antioxidant gene expression, impaired action potential propagation, reduced cell–cell contact, and loss of sarcomere content and organization.^25,48,52,63,64^ Altogether, these findings, in addition to the technical limitations associated with viability and contractility assays in constructs, indicate that the assessment of preservation-associated damage necessitates a multiparameter approach that focuses on construct recovery beyond the mere live and dead and beating rate data.

Indeed, complementing our viability and contractility data with metabolite analyses revealed that the metabolism of HTS-preserved cTE constructs was initially decreased by 40%, but fully recovered at D7 + 3 (Fig. 4A). This was also corroborated by a small increase in glucose consumption and lactate production between D7 + 1 and D7 + 3 (Fig. 4B and C). Conversely, fast frozen samples exhibited an initial sharp reduction (almost 80%) in redox activity (Fig. 4A) combined with negligible glucose consumption and lactate production shortly after rewarming with only a partial recovery at D7 + 7 (Fig. 4B and C). Of note, glucose is also utilized by hfCFs as a substrate. Considering the lack of beating cells, these data suggest that the increased consumption of glucose for fast frozen constructs may be linked to hfCFs proliferation. In the clinic, enhanced lactate levels in the blood are typically taken as a marker for acute heart damage. However, in this case, lactate is released as a by-product of glucose consumption, a finding also observed in the CTRL constructs.^65–67^ An initial decrease in metabolism and its subsequent improvement might suggest that allowing a longer recovery time would result in total functional recovery. Glucose, as well as other substrates are consumed by the hiPSC-CMs, mainly to provide energy for contraction.^68^ Troponin (T and I), one of the main components of the sarcomeres, is released upon cell damage and is commonly used in the clinic as a biomarker for MI and other forms of cardiac injury.^69^ Immunofluorescence revealed that HTS preserved- and, to a larger extent fast frozen constructs, had an appreciable reduction in the number of troponin-T expressing cells (Fig. 5A). In addition, quantification of troponin-I in the conditioned media of cTE constructs revealed a 44-fold and 15-fold increase for fast frozen- and HTS samples, respectively, compared to controls at D7 + 1 (Fig. 5B). The release of cardiac troponin-I in the plasma happens more quickly and reaches much higher levels.^70,71^ At the cardiomyocyte level, the exocytosis of unbound cardiac troponin I is associated with damaged mitochondria and sarcomere protein removal and is enhanced in cardiomyocytes during stress.^72^ Taken together these data suggest that reduced function and metabolism in HTS-preserved constructs might be linked to cells rearranging their sarcomere structures and loss of cell–cell contacts. The latter could be partially reversed by allowing cTE constructs to recover in culture. Whereas in fast frozen constructs, the significant decrease in cardiac troponin T positive cells might indicate irreversible damage.

The development of a polymeric scaffold with arranged fibers was associated with improved cardiac cell arrangement and properties comparable to the native myocardium.^73^ Additionally, upon implantation, cTE constructs need to withstand the continuous contractions and distensions of the heart, which can result in altered mechanical properties induced by irreversible crystallinity changes that may occur during cryopreservation.^20,53^ Furthermore, rapid temperature shifts in polymer filaments were associated with shrinking and consequent cell detachment and ultimately apoptosis.^20^ In this study, the rapid cooling/warming of the PCL filaments did not result in (irreversibly) altered surface topography, or statistically significant differences in crystallinity, glass and melting temperature, or mechanical properties (Fig. 7). Ultimately, ensuring suitable mechanical properties is important for successful implantation and long-term retention. The cTE model used in this study demonstrates how small changes in crystallinity (Fig. 7B) are overpowered by the geometry/design of the scaffold according to the uni-axial direction (Fig. 7C). Our results show no significant adverse effects of preservation on the polymer scaffolds, providing evidence that scaffold-free constructs of similar thickness would not yield different results. While increasing construct thickness could enhance the therapeutic effect, preserving larger cTE constructs is expected to be increasingly difficult, as bigger tissues often suffer from uneven freezing and thawing. Instead, thin constructs, such as the ones used in this study, bypass these issues and still hold clinical significance as stacking of thin constructs is possible and already applied in an ongoing clinical trial.^74^

Altogether, our results suggest that cryogenic preservation induces severe and irreversible damages to cTE constructs, which display signs of hiPSC-CM dysfunction and death. DMSO toxicity may play a role in the decay of the cTE construct. Additionally, it is conceivable that the low thermal strain of the construct in combination with uneven cooling, results in ECM contraction/expansion and (limited) ice nucleation. This would negatively affect cell–cell and cell–matrix interactions, contributing to increased hiPSC-CM death.^46,75^ As cell death is permanent, cryopreservation technology needs further improvements to achieve preserved post-storage cTE construct viability and functionality. Several approaches could be applied focusing on both the biology and methodology. First, toxic CPAs may be (partially) substituted by non-toxic alternatives, such as ice-binding proteins and other materials that inhibit recrystallization and/or promote nucleation near 0 °C. Second, the field of cryopreservation is moving towards the use of CPA cocktails with higher CPA concentrations (*e.g.*, DP6, VS55, and M22) and ultra-fast cooling and rewarming rates (>10^7^ °C min^−1^) to entirely avoid the formation of ice crystals. This method, referred to as vitrification, allows for the samples to acquire a glassy state without ice nucleation, circumventing ice-associated damage to cell–cell junctions and the extracellular matrix.^50,75–78^ Recently, rat kidney vitrification was successfully performed using perfused iron oxide nanoparticles to uniformly heat the sample, resulting in the successful transplantation of the preserved kidney.^79^

In turn, HTS-preserved cTE constructs were correlated with high viability and fast metabolic recovery, with better preservation of hiPSC-CM sarcomere ultrastructure. However, in hypothermia time does not “stop”, but it merely slows down. Therefore, HTS provides a good platform for short-term preservation, extending the time between production and application, allowing construct transport.

## Conclusion

Overall, the results of this study provide insights into the preservation-associated changes to cTE constructs and suggest that HTS is a promising solution for their short-term preservation. Further studies are required to optimize promising long-term preservation techniques, such as fast freezing and vitrification, as well as to evaluate their long-term effects on the cTE constructs. To conclude, our findings provide a basis for the development of efficient preservation techniques, ultimately improving the accessibility and potential benefits of cTE approaches for patients suffering from heart diseases.

## Author contributions

Conceptualization (JJ, LvL, AvM), data curation (NC, JJ, MJA, VSP), formal analysis (NC, JJ, MJA, GCS, MV), investigation (NC, JJ, MJA, GCS, MV, ID), methodology (NC, JJ, MJA, TV, MS, MDC, AvM), writing – original draft (NC, JJ, MA, VSP, AvM), writing – review & editing (JJ, NC, MJA, ID, TV, PAD, MS, IKV, JM, LvL, JPGS, VSP, AvM), funding acquisition (AvM, JPGS, PAD, JM, TV, LvL, IKV, VSP), resources (TV, JM, MDC), software (NC, MA, GCS, VSP), project administration (AvM, JPGS), supervision (AvM, VSP, JPGS, MDC, JM, TV).

## Conflicts of interest

There are no conflicts of interest to declare.

## Supplementary Material

BM-012-D3BM01908J-s001

BM-012-D3BM01908J-s002

BM-012-D3BM01908J-s003

BM-012-D3BM01908J-s004

BM-012-D3BM01908J-s005

BM-012-D3BM01908J-s006

BM-012-D3BM01908J-s007

BM-012-D3BM01908J-s008

BM-012-D3BM01908J-s009

BM-012-D3BM01908J-s010

BM-012-D3BM01908J-s011

BM-012-D3BM01908J-s012

BM-012-D3BM01908J-s013

BM-012-D3BM01908J-s014

BM-012-D3BM01908J-s015

BM-012-D3BM01908J-s016

BM-012-D3BM01908J-s017
