# Supplementary material for: Hypothermic and cryogenic preservation of cardiac tissue-engineered constructs
Source: Biomater Sci. 2024 Jun 24;12(15):3866–81. doi: 10.1039/d3bm01908j (PMC11265564; doi:10.1039/d3bm01908j)
Supplement: BM-012-D3BM01908J-s001 [file BM-012-D3BM01908J-s001.pdf]

Electronic Supplementary Material (ESI) for Biomaterials Science.
This journal is © The Royal Society of Chemistry 2024

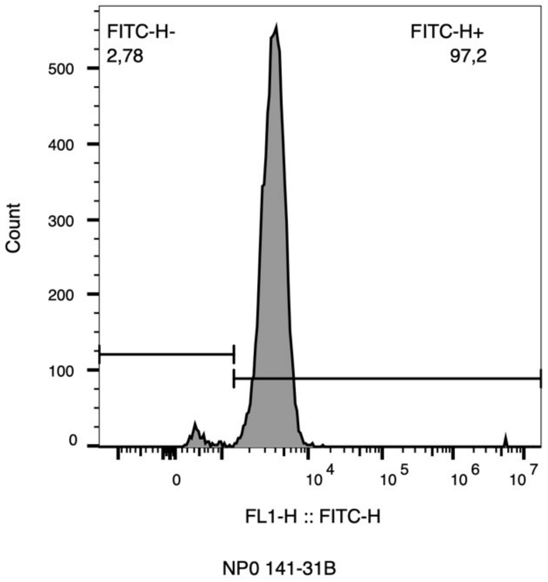

Figure S1: FACS analysis of GFP-tagged a-actinin to determine NP0 141-31B iPSC-CM purity.

Supplementary Information

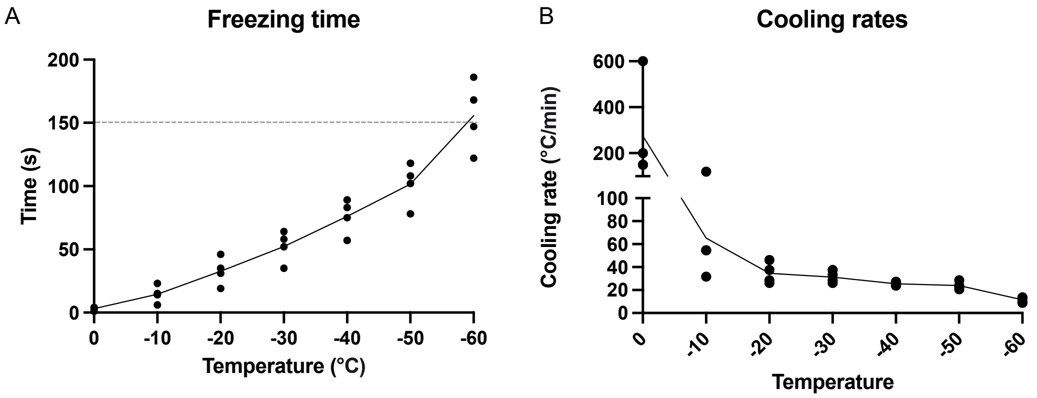

Figure S2: Determination of cooling rates during fast freezing to -60°C. A) The sample temperature over time, measured with a submerged probe from a digital thermometer in the freezing solution, indicates rapid freezing. Fast frozen constructs were immersed in isopropanol for 150 s (marked by the gray dotted line). B) The measured cooling rate was slowest between -50°C and -60°C (averaging 11.44°C/minute), still 10 times faster than the freezing rate applied to slow frozen constructs.

Figure S3: Overview of image acquisition. A) Graphical schematic representation of image locations and depth. B) Overview of the first slice, middle slice, last slice, and maximum projections of LIVE/DEAD z-stacks taken from different experimental groups and at different timepoints. Channels are overlaid. Scale bar = 100 µm

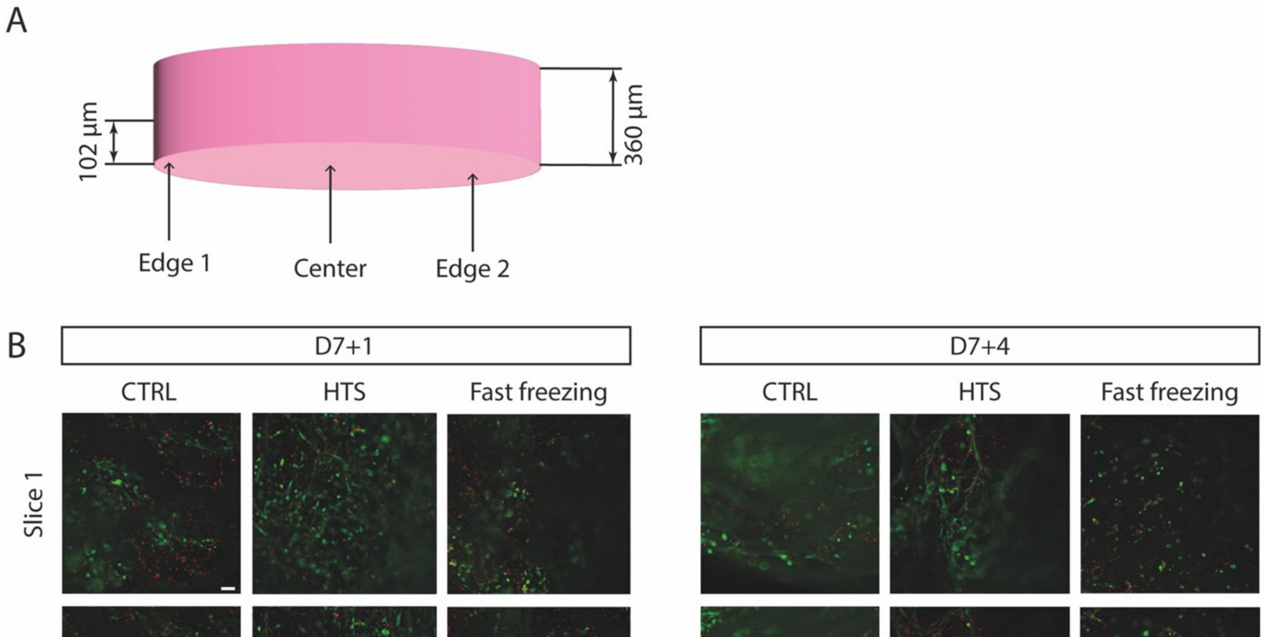

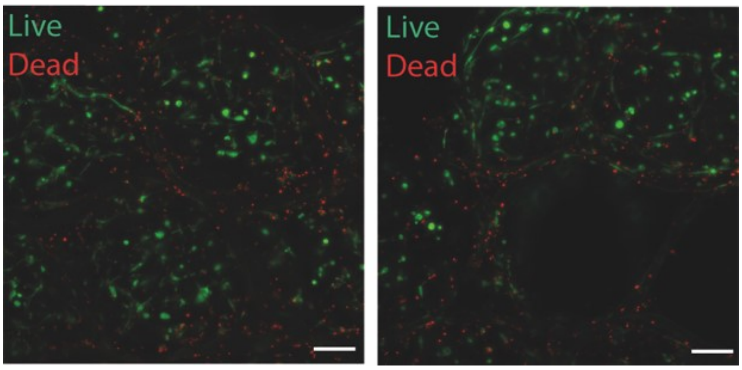

Figure S4: Live/dead imaging (D7+4) of fast frozen cTE constructs containing D21 iPSC-CMs. Living cells, (green/calcein-AM positive), and dead cells (red/ethidium homodimer-1 positive). Channels are overlaid. Scale bar = 150 µm. Representative images are shown.

Table S1: Overview of culture media used during this study.

	**Base Media**	**Supplements**
hfCFb culture media	DMEM ([+] 4.5 g D-Glucose, L-Glutamine, [-] Pyruvate) (Gibco, 41965-039)	10% Fetal bovine serum (FBS) (Biowest, S1810-500)<br>1% Penicillin-Streptomycin (Gibco, 15-140-122)
Construct culture media	DMEM ([+] 4.5 g D-Glucose, L-Glutamine, [-] Pyruvate) (Gibco, 41965-039)	10% KnockOut™ serum replacement (KOSR) (Thermo Fisher Scientific, 10828028)<br>2% B27® (Gibco, 17504-001)<br>1% Penicillin-Streptomycin (Gibco, 15-140-122)

Table S2: Overview of preservation media.

Name	Composition
Rokepie (Rp)	DMEM ([+] 4,5 g/L D-Glucose, L-Glutamine, [-] Pyruvate) (Gibco, 4965-039) + 5% Rokepie-FD01® (Rokepie, RS.01.10) + 1% Penicilin/streptomycin (Gibco, 15-140-122)
HypoThermosol® (HTS)	HypoThermosol® (HTS) (BioLife solutions, 101102)
University of Wisconsin (UW) solution	Costorsol™ (PreservationSolutions)
Standard freezing media	Fetal bovine serum (FBS) (Biowest, S1810-500) + 10% Dimethyl Sulfoxide (DMSO) (Sigma-Aldrich, D2650)

Table S3: Antibodies list used for fluorescent immunohistochemistry experiments.

**Antibody host and target**	**Reference**
Rabbit anti-cardiac Troponin T	Abcam ab45932
Mouse anti-α-actinin	Merck A7811, USA
AlexaFluor goat anti-rabbit 568	Invitrogen A11036
AlexaFluor goat anti-mouse 488	Invitrogen A11029
Hoechst 33342	Invitrogen H3570

List of supplementary videos

Video S1 CTRL D7+4 BR

Video S2 HTS D7+4 BR

Video S3 CRYO Slow Freezing D7+4 BR

Video S4 CRYO Fast Freezing D7+4 BR

Video S5 D10 iPSC-CMs pre-preservation

Video S6 D14 iPSC-CMs pre-preservation

Video S7 D21 iPSC-CMs pre-preservation

Video S8 d14 iPSC-CMs D7+3 CTRL

Video S9 d14 iPSC-CMs D7+3 HTS

Video S10 d14 iPSC-CMs D7+3 Fast Freezing

Video S11 d21 iPSC-CMs D7+3 CTRL

Video S12 d21 iPSC-CMs D7+3 HTS

Video S13 d21 iPSC-CMs D7+3 Fast Freezing

Video S14 CTRL D7+4 CAL520

Video S15 HTS D7+4 CAL520

Video S16 CRYO Fast Freezing D7+4 CAL520
